# Case Report: Analysis of Circulating Tumor Cells in a Triple Negative Spindle-Cell Metaplastic Breast Cancer Patient

**DOI:** 10.3389/fmed.2021.689895

**Published:** 2021-06-24

**Authors:** Tania Rossi, Michela Palleschi, Davide Angeli, Michela Tebaldi, Giovanni Martinelli, Ivan Vannini, Maurizio Puccetti, Francesco Limarzi, Roberta Maltoni, Giulia Gallerani, Francesco Fabbri

**Affiliations:** ^1^Biosciences Laboratory, IRCCS Istituto Romagnolo per lo Studio dei Tumori (IRST) “Dino Amadori”, Meldola, Italy; ^2^Department of Medical Oncology, IRCCS Istituto Romagnolo per lo Studio dei Tumori (IRST) “Dino Amadori”, Meldola, Italy; ^3^Unit of Biostatistics and Clinical Trials, IRCCS Istituto Romagnolo per lo Studio dei Tumori (IRST) “Dino Amadori”, Meldola, Italy; ^4^Scientific Directorate, IRCCS Istituto Scientifico Romagnolo per lo Studio dei Tumori (IRST) “Dino Amadori”, Meldola, Italy; ^5^Azienda Unità Sanitaria Locale Imola, Imola, Italy; ^6^Pathology Unit, Morgagni-Pierantoni Hospital, Forlì, Italy; ^7^Healthcare Administration, IRCCS Istituto Romagnolo per lo Studio dei Tumori (IRST) “Dino Amadori”, Meldola, Italy

**Keywords:** metaplastic breast cancer, circulating tumor cells, next generation sequencing, copy number aberration, metastasis, liquid biopsy

## Abstract

Circulating tumor cells (CTCs) are a rare population of cells found in the bloodstream and represent key players in the metastatic cascade. Their analysis has proved to provide further core information concerning the tumor. Herein, we aim at investigating CTCs isolated from a 32-year-old patient diagnosed with triple negative spindle-shaped metaplastic breast cancer (MpBC), a rare tumor poorly responsive to therapies and with a dismal prognosis. The molecular analysis performed on the primary tumor failed to underline effective actionable targets to address the therapeutic strategy. Besides the presence of round-shaped CTCs, cells with a spindle shape were present as well, and through molecular analysis, we confirmed their malignant nature. This aspect was coherent with the primary tumor histology, proving that CTCs are released regardless of their morphology. Copy number aberration (CNA) profiling and variant analysis using next-generation sequencing (NGS) showed that these cells did not harbor the alterations exhibited by the primary tumor (*PIK3CA* G1049A mutation, *MYC* copy number gain). However, despite the great heterogeneity observed, the amplification of regions involved in metastasis emerged (8q24.22–8q24.23). Our findings support the investigation of CTCs to identify alterations that could have a role in the metastatic process. To the best of our knowledge, this is the first examination of CTCs in an MpBC patient.

## Introduction

Among all the breast malignancies, metaplastic breast cancer (MpBC) accounts for <1% and has a dismal prognosis, worse than the other BC types. Pathologically, MpBCs are ductal carcinomas composed by one or more cell populations that have undergone metaplastic transformation into a non-glandular pattern, leading to the presence of epithelial (e.g., squamous cells) and sarcomatous (e.g., chondroid, spindle cell, and osseous) elements. The World Health Organization (WHO) further divides MpBCs in subgroups, resulting in a plethora of chemorefractory and aggressive MpBC variants (1–3).

Despite the paucity of MpBC cases, some studies in literature have detected epithelial-to-mesenchymal transition (EMT), phosphoinositide 3-kinase (PI3K) signaling, epidermal growth factor receptor (EGFR) signaling, and others as the major altered pathways in this disease (3). Nevertheless, the lack of actionable targets remains a matter of concern, and conventional regimens of chemotherapy mainstay are the gold standards for treatment together with surgery and radiation therapy (4, 5). However, the poor survival and the high recurrence rates further emphasize the inadequacy of the available treatment options and the imperative need to individuate appropriate therapeutic strategies.

Genetic and phenotypic heterogeneity is a hallmark of MpBC and has important reflections for cancer treatment as the presence of multiple clones may hide cells responsible for relapse (6, 7). In this context, the characterization of circulating tumor cells (CTCs), a rare population of cells considered as pro-metastasis precursors (8), may be helpful in the unraveling of tumor heterogeneity (9).

We report a case of a patient diagnosed with triple negative spindle-cell MpBC for which molecular analysis of the primary tumor failed to highlight valid actionable alterations. We decided to characterize CTCs at both morphological and molecular levels, as they may bring out new alterations to be explored. To the best of our knowledge, this is the first examination of CTCs in a MpBC patient.

## Case Presentation

**Figure 1:**
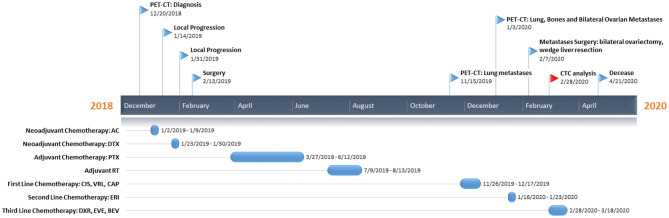
Patient's timeline. At the top, we reported the milestones of her clinical course, while on the bottom the treatment regimens administered to the patient. PET-CT, positron emission tomography-computed tomography; AC, adryamicin-cyclophosphamide; DTX, docetaxel; PTX, paclitaxel; CIS, cisplatin; VRL, vinorelbine; CAP, capecitabine; ERI, eribulin; DXR, doxorubicin; EVE, everolimus; BEV, bevacizumab; CTC, circulating tumor cell.

Here we report the case of a 32-year-old patient (Figure 1) who presented, on December 20, 2018, during breastfeeding, a clinical onset of a right breast lump. Ultrasound-guided core biopsy of this right breast mass was performed with histological diagnosis of metaplastic spindle-cell infiltrating carcinoma of the breast, estrogen receptor (ER) = 0%, progesterone receptor (PgR) = 0%, HER2-neu negative (score 0), and Ki-67 = 90%. She had a past history of Crohn's disease, at the time of MpBC diagnosis during treatment with mesalamine. Positron emission tomography-computed tomography (PET-CT) revealed a 40-mm lesion in the right breast without bone or visceral involvement. In January 2019, treatment began with neoadjuvant chemotherapy (NAC) with adryamicin (60 mg/m^2^) and cyclophosphamide (600 mg/m^2^) intravenous for one cycle. Due to local progression, NAC was switched to docetaxel for one cycle (January 23, 2019), but the patient experienced further local progression.

**Figure 2:**
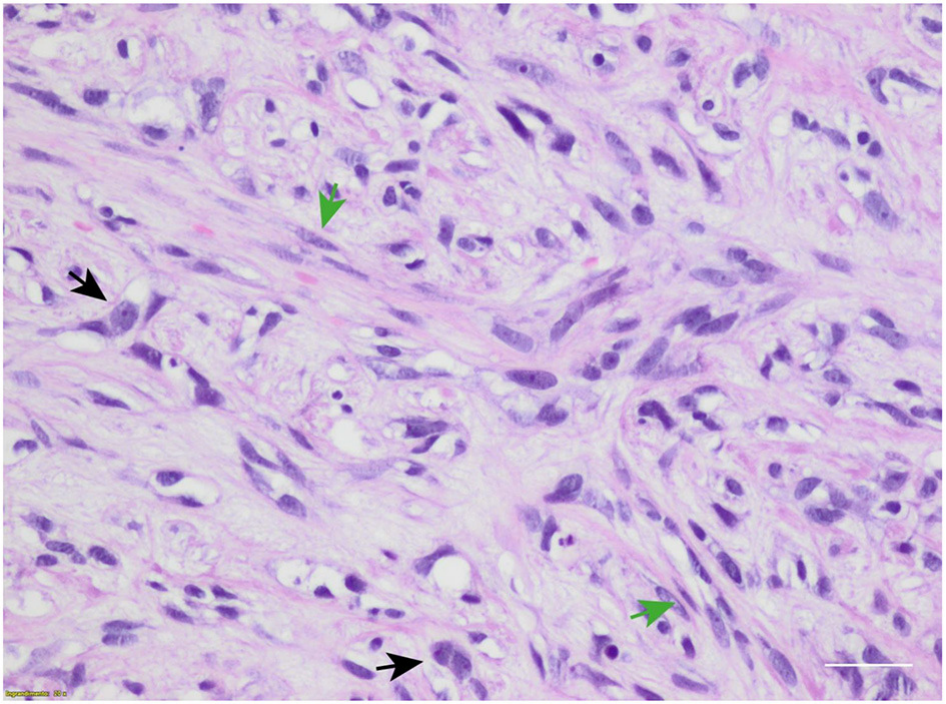
Microscopic hematoxylin and eosin photograph of metaplastic breast carcinoma (MpBC) with coexisting spindled (green arrows) and oval (black arrows) cells. Scale bar: 50 μm.

In February 2019, she underwent right mastectomy with axillary node dissection; the histopathology exam describes a lesion of 65-mm maximum diameter, ypT3 ypN0 M0, ER = 0%, PgR = 0%, HER2-neu negative (score 0), and Ki-67 = 90%. The microscopic photograph (10 × magnification) of hematoxylin and eosin staining of the resected tumor is reported in Figure 2. On immunostains, the tumor cells were strongly positive for vimentin and showed weak positivity for p63. Cytokeratins (AE1/AE3 clone) and E-cadherin were positive in scattered cells. Moreover, CAM5.2, calponin, SMA, GATA-3, ALK, ER, PR, and Her2-neu were negative. Expression of programmed death-ligand 1 (PD-L1) was <1%.

From March to June 2019, she received adjuvant weekly paclitaxel (80 mg/m^2^) for 12 cycles. From July to August 2019 right chest radiotherapy (total dose 50 Gy) was performed.

In November 2019, PET-CT scan revealed the presence of a 40 × 37-mm lung lesion and other sub-centimeter bilateral lung nodules. Further analyses on primary tumor revealed no *BRCA1*/*BRCA2* alterations.

From November to December 2019, she received two cycles of cisplatin (60 mg/m^2^; day 1), vinorelbine (20 mg/m^2^; days 1 and 3), and capecitabine (500 mg thrice a day).

In January 2020, PET-CT showed lung, bone, and bilateral ovarian progression. The NGS Oncomine Focus Assay (Thermo Fisher Scientific) on the primary tumor exposed the G1049A *PIK3CA* mutation and amplification of the *MYC* locus (copy number: 26 copies).

In January 2020, she received two cycles of eribulin (1.23 mg/m^2^), and in February, she underwent bilateral ovariectomy and wedge liver resection. The histopathology exam described triple negative metaplastic BC metastases. Moreover, several subcutaneous metastases on the scalp, neck, and chest arise, other than bilateral lung nodules. After multidisciplinary meeting, in consideration of the absence of valid therapeutic alternatives, physicians decided to start an “off-label” treatment regimen: doxorubicin (30 mg/m^2^) plus bevacizumab (15 mg/kg every 3 weeks) plus everolimus (7.5 mg daily) (10).

In February 2020, she started the first cycle (without bevacizumab due to recent surgery), with a clinically stable disease, improvement on pain, and reduction of all subcutaneous nodules. Before chemotherapy administration, CTC investigation was performed.

In March 2020, she received the second cycle (including bevacizumab). She had a clinical benefit in terms of the disappearance of most subcutaneous metastases, no pain, and a good quality of life until April 2020 when she complained of fever, cough, and low blood pressure. Therefore, she was hospitalized for the appropriate treatment with antibiotics and steroids without benefit, and she died on April 21, 2020 due to respiratory failure.

## Isolation of Circulating Tumor Cells and Analyses

**Figure 3:**
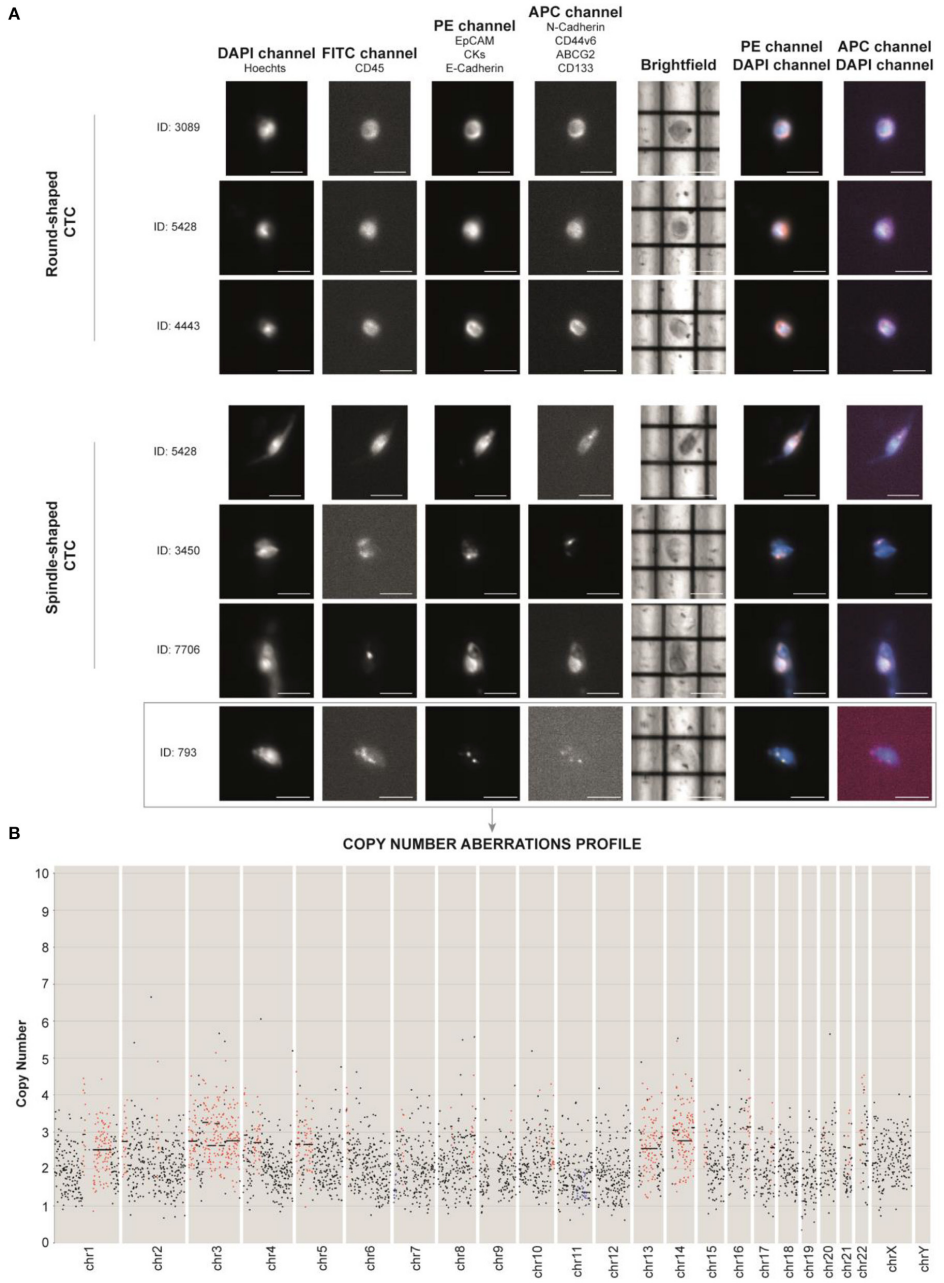
**(A)** DEPArray images of the most representative single circulating tumor cells (CTCs) of the patient based on their shape (round- and spindle-shaped). The DAPI channel was used for nuclear staining using Hoechst 33342, PE channel for epithelial tag [anti-EPCAM, anti-cytokeratins (CKs) and anti-E-cadherin antibodies], and APC channel for mesenchymal tag (anti-N-cadherin, anti-CD44v6, anti-ABCG2, and anti-CD133 antibodies). Anti-CD45 (FITC channel) was used for CTC negative selection (Supplementary Figure 1). Scale bar: 30 μm. **(B)** Profiling of the CTC ID: 793 reveals the presence of aberrant regions, consistent with tumor nature. Chromosomes (Chr) and number of copies are reported along the *x*- and *y*-axis, respectively. Black dots in the figure represent chromosome regions with a normal diploid copy number. Conversely, red dots and blue dots indicate, respectively, significant copy number gains (copies > 2) and losses (copies <2), called by Control-FREEC (11).

To investigate the features of CTCs, approximately 9 ml of peripheral blood was collected in a PAXGene Blood ccfDNA tube before the administration of the off-label therapy. CTCs were enriched from whole blood by immunomagnetic negative selection. In order to identify the highest number of CTCs, we opted for antibody cocktails for the detection of each phenotype, instead of a single target for each channel. EpCAM, CKs, and E-cadherin antibodies were used to identify epithelial phenotype (phycoerythrin, PE channel), and N-cadherin, ABCG2, CD44v6, and CD133 were used to identify stem/mesenchymal phenotype (allophycocyanin, APC channel). Hoechst 33342 (DAPI channel) was used for nuclear staining and anti-CD45 Alexafluor488 antibody (FITC channel) as leukocyte marker for CTC negative selection. CTC identification and analysis were performed by DEPArray NxT platform (Figure 3A). To set up the auto-fluorescence signal detected in FITC channel, we used MCF7 cell line (CD45–) and leukocytes (CD45+) (Supplementary Figure 1).

We identified non-canonical cells positive for both epithelial and stem/mesenchymal targets with a spindle-shaped morphology (*n* = 14) and CTCs with a round-shaped morphology (*n* = 184). CTC clusters (*n* = 5) were present as well (Supplementary Figure 2). Due to the high amount of debris into the sample, we successfully sorted one single spindle-shaped cell (ID: 793) and two 10-CTC pools (Pool 1 and Pool 2).

Next, we aimed at assessing the molecular characteristics of the MpBC patient's CTCs isolated through DEPArray and to establish the nature of the spindle-shaped cell. To do this, we massively amplified the genome of the samples using Ampli1 WGA kit (Menarini-Silicon Biosystems) to obtain evaluable genetic material, then we proceeded with library construction and sequencing for copy number aberrations (CNAs) and single nucleotide variant (SNV) analyses.

For CNAs, libraries were prepared using the Ampli1 LowPass kit for Ion Torrent (Menarini-Silicon Biosystems).

After Ion 520 chip loading was performed on Ion Torrent Chef (Thermo Fisher Scientific), sequencing was carried out on an Ion S5 System (Thermo Fisher Sc.), and CNAs were called with Control-FREEC (11). Through this technique, we were able to unequivocally establish the tumor nature of the unconventional spindle-shaped CTC (ID: 793), since it was characterized by an aneuploid genome with an altered CNA profile (Figure 3B). Through intersection bioinformatic analysis, we observed that the greatest part of the entire genome was not comparable among the three samples, suggesting high heterogeneity levels. We detected only three mutual aberrant regions (4p16.1, 8q24.22–8q24.23, and 22q12.3) shared among the samples, which were always in gain. The genes within the mentioned regions are reported in Supplementary Table 1. We did not observe the gain of *MYC* gene, which was observed in the primary tumor molecular characterization. Moreover, after whole genome amplification, we assessed the mutational status of 60 cancer-related genes. Libraries were prepared using the Ampli1 OncoSeek kit (Menarini Silicon Biosystems) and run on a 300-cycle V2 cartridge on the miSeq instrument (Illumina Inc.). We did not observe the *PIK3CA* mutation G1049A, which emerged at primary tumor NGS analysis. The single CTC 793 harbored the homozygous *RET* I602V and heterozygous M249V variant of gene *MAP2K1*. Interestingly, we also found a heterozygous synonymous C50C variant of *TP53* that, although not responsible for the amino acid change in protein structure, may be associated with gene expression regulation as it occurs in the noncoding exon 1 (12). Concerning the 10-CTC pools, in Pool 1, we found the variants *KRAS* S17I (frequency 80%), *PIK3CA* W498C (20%), *PTEN* A34T (16%), *MET* T835N (14%), *PIK3CA* L1026I (14%), *SMAD4* Q249R (10%), *KIT* G534V (10%), *MAP2K1* G131A and N122K (10%), and *PTEN* M35T (10%). Pool 2 harbored the *HRAS* frameshift deletion I46fs (20%) and the non-synonymous SNV of *RB1* R556G (10%). None of the detected variants were already described as pathogenic in literature, and no variants were identified in common among the samples. However, frequencies of some alterations found in samples Pool 1 and Pool 2 reveal that more than one cell within each pool harbor certain mutations.

## Discussion

Here, we report a case of a 32-year-old patient diagnosed with triple-negative spindle-cell MpBC, a rare disease with marked tendency to metastasize to secondary organs. Because of the paucity of cases, few is known about the molecular mechanisms underlying its aggressiveness, and no druggable targets have been identified yet.

Investigation of the primary tumor performed through an NGS-based approach detected alterations that are not uniquely ascribable to MpBC and failed to underline effective actionable targets to address the therapeutic strategy. Indeed, *MYC* copy number gain (13, 14) and *PIK3CA* mutation (15, 16) are widely described in the literature to be involved in many cancer types, including BC (17, 18). Herein, by exploiting a liquid biopsy approach, we emphasize that the characterization of CTCs, rare cells with a crucial role in the metastatic cascade, could be worthwhile to guide the investigation of the molecular mechanisms underlying rare tumors. However, this study has some weaknesses. First, due to the high amount of debris in the sample, it was not possible for us to recover, for downstream analysis, all the CTCs found. Moreover, the primary tumor and metastatic tissue specimens were not available, making impossible the comparison with CTCs, except for clinical reports. Conversely, to the best of our knowledge, this is the first investigation of CTCs in an MpBC case and could have a strong impact in the improvement of personalized medicine in the future. In fact, the investigation of CTCs has the potential to unmask the intra-tumor heterogeneity of MpBC, revealing the presence of under-represented clones from the primary tumor. Simultaneously, longitudinal molecular profiling of CTCs could reveal acquired resistance mechanism, helping to address the therapeutic strategy (19).

Our analysis revealed a high count of CTCs expressing both epithelial and mesenchymal markers in the peripheral blood of the MpBC patient. CTC enumeration and evaluation have been performed in a delayed period after removal of the primary tumor. Therefore, in the absence of their major source, CTCs are likely to come from secondary homing sites such as bone marrow or other occult niches (9, 20).

The first criterion concerned the different morphology of CTCs retrieved in the bloodstream of the patient. Along with the canonical round CTCs, we found unconventional cells characterized by a spindle morphology, whose nature was confirmed to be cancerous due to the presence of CNAs along the genome. A study by Yu et al., conducted on non-metaplastic BC patients, demonstrates that besides the presence of round-shaped CTCs with unconventional morphology associated with EMT initiation, therapy failure and tumor progression were present as well (21). However, it is not clear whether the presence of both round- and spindle-shaped CTCs in this MpBC case assumes the same meaning as in non-metaplastic BC, or is consistent with tumor histology solely. Indeed, the cytopathology of CTCs detected in this MpBC case is coherent with primary tumor histology, in which oval and spindle-shaped cells were shown to coexist. Thorough analysis on the metastatic site specimens would provide further information about the role of CTC morphology in MpBC tumor progression.

Concerning molecular analyses, we found that CTCs did not harbor primary tumor-specific alterations (*MYC* copy number gain and G1049A *PIK3CA* mutation). In addition, CTC samples had discordant CNAs and SNVs compared with each other, suggesting molecular heterogeneity as well. These results are consistent with the presence of circulating heterogeneous subclones with a potential role in tumor progression and resistance to therapies. Accordingly, in the HR-positive metastatic BC patient, CTCs could circulate as heterogeneous subclones and harbor molecular alterations that could drive to different mechanisms of resistance to endocrine therapies (22). Together with our data, these findings support the need to increase the application of CTCs in the clinical practice to gain further complementary information concerning the tumor evolution, including therapy resistance.

CNA profiling revealed the existence of three amplified genomic regions (4p16.1, 8q24.22–8q24.23, and 22q12.3) shared among the CTC samples. Among these, region 8q24.22–8q24.23 turned out to be highly attractive.

Amplification of chromosome 8q regions has been described in numerous cancer types, such as hepatocellular carcinoma (23), gastric cancer (24), clear cell renal carcinoma (25), and BC (26). In particular, in BC, 8q is considered a hotspot site of amplification associated with unfavorable prognosis (27) and, accordingly in our study, poor response to NAC (28), in part due to the location of c-myc locus in 8q24.1, near 8q24.22–8q24.23. However, the tendency to drive metastasis is not only imputable to *MYC* solely, but other genes have been hypothesized to enhance the metastatic process, as reported by Han and collaborators (28). Accordingly, investigation of CTCs revealed that several cancer-associated genes were found altered, but not *MYC*.

For instance, *WISP1* codifies for WNT1-inducible signaling pathway protein 1 (WISP1/CCN4), a member of the CCN family that acts as an oncogene in BC. It has been shown to stimulate EMT and metastasis, and to modulate the expression of the tumor suppressor N-myc downstream-regulated gene 1 (NDRG1) in BC cell lines. This modulation occurs through the NDGR1 gene promoter, which is located within the 8q24.23 site (29). However, the role of this tumor-suppressor protein is controversial, as recent data highlighted that NDRG1 expression is a predictor of worse prognosis in inflammatory BC patients receiving adjuvant radiotherapy (30). Both *WISP1* and *NDGR1* loci are included within the regions that we found in gain in all the CTC samples.

Another gene located in the 8q22.2 locus that recently emerged as a metastasis driver is the Otoconin 90 (*OC90*) gene. Although it is normally expressed in the cochlea for appropriate otolith development, its expression and gene amplification were observed in different cancer types, such as breast, prostate, and lung cancer. In TNBC cell lines, *OC90* overexpression was shown to increase invasion, and knockdown reduced cellular viability and invasiveness (31).

Taken together, despite high levels of heterogeneity, we observed that the analysis of CTCs at the molecular level can potentially drive to the discovery of chromosomal regions that may have a role in the metastatic cascade. In the future, this aspect could be helpful to deepen the knowledge regarding MpBC and to address new therapeutic strategies.

## Data Availability Statement

The datasets presented in this article are not readily available for ethical and privacy reasons. Requests to access the datasets should be directed to the corresponding author.

## Ethics Statement

Ethical review and approval was not required for the study on human participants in accordance with the local legislation and institutional requirements. The patients/participants provided their written informed consent to participate in this study. Written informed consent was obtained from the individual(s) for the publication of any potentially identifiable images or data included in this article.

## Author Contributions

TR, MPa, GM, IV, GG, and FF contributed to the conceptualization and design of the study. TR and MPa wrote the first draft. DA and MT performed bioinformatic analysis. TR, MPa, MPu, FL, RM, and GG contributed to data collection. GG and FF reviewed the final draft before submission. All authors contributed to the manuscript revision, and read and approved the submitted version.

## Conflict of Interest

The authors declare that the research was conducted in the absence of any commercial or financial relationships that could be construed as a potential conflict of interest.
