# Supplementary material for: Case Report: Analysis of Circulating Tumor Cells in a Triple Negative Spindle-Cell Metaplastic Breast Cancer Patient
Source: Front Med (Lausanne). 2021 Jun 24;8:689895. doi: 10.3389/fmed.2021.689895 (PMC8264184; doi:10.3389/fmed.2021.689895)

Supplementary Material

Supplementary Figure 1.

Set up of auto-fluorescent signal detected on FITC channel. Leukocytes and MCF7 cells were used, respectively as positive (CD45+) and negative (CD45-) controls. The CD45 specific signal in leukocytes is well contrasted and localized in the cell membrane. Conversely, auto fluorescence CD45 signal in MCF7 cells presents as diffused and the contrast between background and signal is poor. Scale bar: 30 µm.

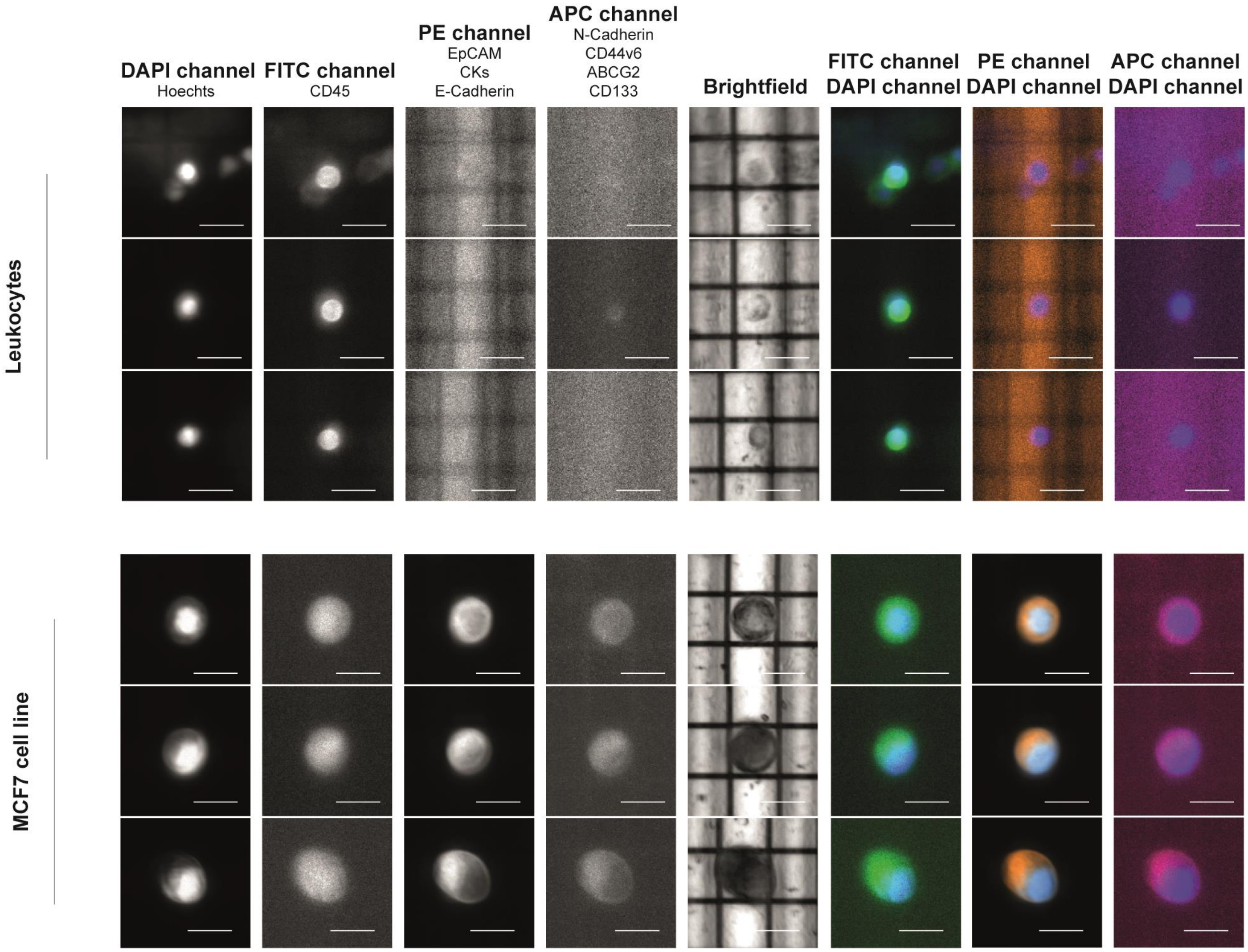

Supplement: Supplementary file 1 [file Image_1.pdf]
