# Supplementary material for: Case Report: Analysis of Circulating Tumor Cells in a Triple Negative Spindle-Cell Metaplastic Breast Cancer Patient
Source: Front Med (Lausanne). 2021 Jun 24;8:689895. doi: 10.3389/fmed.2021.689895 (PMC8264184; doi:10.3389/fmed.2021.689895)

Supplementary Material

Supplementary Figure 2.

DEPArray images of the most representative circulating tumor cell (CTC) clusters observed in the peripheral blood of metaplastic breast cancer (MpBC) patient. Scale bar: 30 µm.

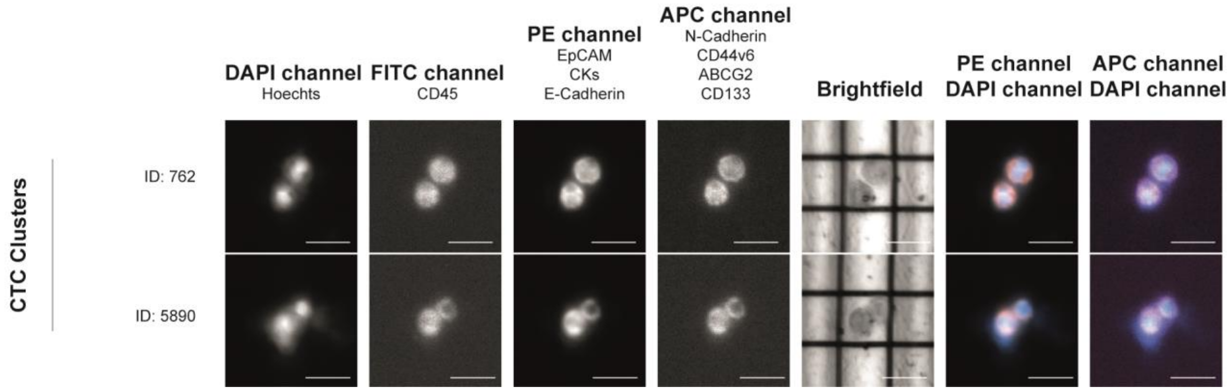

Supplement: Supplementary file 2 [file Image_2.pdf]
