# Supplementary material for: Case Report: Analysis of Circulating Tumor Cells in a Triple Negative Spindle-Cell Metaplastic Breast Cancer Patient
Source: Front Med (Lausanne). 2021 Jun 24;8:689895. doi: 10.3389/fmed.2021.689895 (PMC8264184; doi:10.3389/fmed.2021.689895)
Supplement: Supplementary file 3 [file Table_1.docx]

Supplementary Material

**Supplementary Table 1.**

List of regions commonly altered regions among circulating tumor cell (CTC) samples, and the genes localized within. Chr: chromosome; bp: base pair.

| Chr | Start (bp) | End (bp) | Status | Genes |
| --- | --- | --- | --- | --- |
| 4 | 5.407.073 | 10.266.210 | Gain | *ABLIM2; ACOX3; AFAP1; BLOC1S4; C4orf6; CCDC96; CPZ; CRMP1; DEFB131; DRD5; EVC; EVC2; GPR78; GRPEL1; HMX1; HTRA3; JAKMIP1; KIAA0232; MAN2B2; MRFAP1; MRFAP1L1; PPP2R2C; PSAPL1; S100P; SH3TC1; SLC2A9; SORCS2; TADA2B; TBC1D14; TRMT44; USP17L10; USP17L11; USP17L12; USP17L13; USP17L15; USP17L17; USP17L18; USP17L19; USP17L20; USP17L21; USP17L22; USP17L24; USP17L25; USP17L26; USP17L27; USP17L28; USP17L29; USP17L30; USP17L5; WDR1; WFS1* |
| 8 | 131.407.488 | 133.460.730 | Gain | *ADCY8; EFR3A; HHLA1; KCNQ3; OC90* |
| 8 | 134.145.144 | 139.474.478 | Gain | *FAM135B; KHDRBS3; NDRG1; ST3GAL1; WISP1; ZFAT* |
| 22 | 32.851.872 | 35.369.126 | Gain | *FBXO7; LARGE; SYN3; TIMP3* |
| 22 | 35.369.126 | 37.849.511 | Gain | *APOL1; APOL2; APOL3; APOL4; APOL5; APOL6; C1QTNF6; CACNG2; CSF2RB; CYTH4; EIF3D; ELFN2; FOXRED2; HMGXB4; HMOX1; IFT27; IL2RB; ISX; KCTD17; MB; MCM5; MPST; MYH9; NCF4; PVALB; RAC2; RASD2; RBFOX2; SSTR3; TEX33; TMPRSS6; TOM1; TST; TXN2* |
